# PbE (E = S, Se) Colloidal Quantum Dot-Layered 2D Material Hybrid Photodetectors

**DOI:** 10.3390/nano10010172

**Published:** 2020-01-19

**Authors:** Tom Nakotte, Hongmei Luo, Jeff Pietryga

**Affiliations:** 1 Department of Chemical and Materials Engineering, New Mexico State University, Las Cruces, NM 88003, USA; tnakotte@nmsu.edu; 2 Chemistry Division, Los Alamos National Laboratory, Los Alamos, NM 87545, USA; pietryga@lanl.gov

**Keywords:** colloidal quantum dots, layered 2D materials, photodetectors, infrared, PbSe, PbS, graphene, phosphorene, TMDs

## Abstract

Hybrid lead chalcogenide (PbE) (E = S, Se) quantum dot (QD)-layered 2D systems are an emerging class of photodetectors with unique potential to expand the range of current technologies and easily integrate into current complementary metal-oxide-semiconductor (CMOS)-compatible architectures. Herein, we review recent advancements in hybrid PbE QD-layered 2D photodetectors and place them in the context of key findings from studies of charge transport in layered 2D materials and QD films that provide lessons to be applied to the hybrid system. Photodetectors utilizing a range of layered 2D materials including graphene and transition metal dichalcogenides sensitized with PbE QDs in various device architectures are presented. Figures of merit such as responsivity (*R*) and detectivity (D*) are reviewed for a multitude of devices in order to compare detector performance. Finally, a look to the future considers possible avenues for future device development, including potential new materials and device treatment/fabrication options.

## 1. Introduction and Background

**Figure 1:**
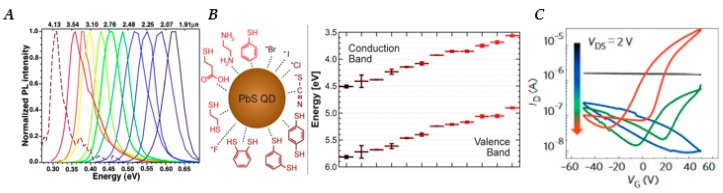
(**A**) Normalized photoluminescence spectra of lead selenide (PbSe) quantum dots (QDs) of varying sizes, as an indication of bandgap tenability (bottom x-axis has units of energy (eV) ranging from 0.30 to 0.65 eV, top x-axis has units of wavelength ranging from 4.13 to 1.91 µm). Reproduced with permission from [13]. Copyright American Chemical Society, 2004. (**B**) Illustration of a lead sulfide (PbS) QD with various types of ligands (left), and the corresponding (by color) conduction and valence band energy levels each ligand produces (right). The positions of the valence and conduction bands of PbS QDs with different ligands are presented in the graph to the right. Beginning with Br^-^ (brown color, lowest band positions) moving clockwise around the schematic of the QD until it reaches benzenethiol (red color, highest band positions). Reproduced with permission from [18]. Copyright American Chemical Society, 2014. (**C**) Transfer characteristics of a 5.9 nm PbSe QD film after solid-state exchange with sodium selenide (Na_2_Se) (black), which removes long oleate ligands and enriches the surface in Se, and subsequently upon lead chloride (PbCl_2_) treatment for durations of 1 h (blue), 6 h (green) and 12 h (red) at 65 °C, which enriches the surface in metal. *I_D_*, drain current; *V_G_*, gate voltage; *V_DS_*, drain–source voltage. Reproduced with permission from [64]. Copyright American Chemical Society, 2014.

Lead chalcogenide (PbE, E = S, Se) quantum dots (QDs) are desirable materials for implementation in photodetectors because of their potential for improving upon current technologies in the near-infrared (near-IR) and mid-IR spectral regions [1,2,3,4]. Improving capabilities in this region is of great consequence for applications in environmental monitoring [5,6,7], motion sensing [8], fiber-optic communications [9,10], X-ray detection [11], and biological imaging [12], where Pb-based QDs have been shown to be less destructive to cells than there Cd-based counterparts when coated with silica. PbE QDs have size-tunable bandgaps from 800 to 4000 nm (1.55–0.31 eV) [13,14], as shown in Figure 1A, and their solution processability allows for facile integration onto both rigid and flexible substrates through spin-coating, dip-coating, and ink-jet printing [4,15]. The ease with which QD solutions can be integrated, as sensitizers for the near-IR wavelength regions into current silicon based technologies, which are based on complementary metal-oxide-semiconductor (CMOS) architectures make them ideal candidates for further study [16]. Along with the profound effect size has on the properties of QDs, altering the surface chemistry via ligand exchange or other post-deposition treatments can also greatly alter the physical and electronic properties of QD films [17,18,19,20,21,22,23], affecting properties such as carrier type and the smoothness of the QD film. Quality lead sulfide (PbS) QD photodetectors were initially reported in 2005 by Sargent, et al. [24]; during the years since then many efforts have been made to understand, and improve QD devices [17,25,26,27,28,29]. 

Successfully absorbing incident photons and collecting the photogenerated carriers from the QD film is paramount to fabricating QD-based photodetectors. PbE QDs are strong absorbers that efficiently convert incident photons into electron-hole pairs (excitons), even displaying carrier multiplication, which is the generation of multiple excitons per single absorbed photon with an energy of two or more times greater than the bandgap of the QD [30,31,32]. The discovery of carrier multiplication in PbE QDs created momentum for the QD solar cell path [33,34,35,36,37,38,39], which led to many studies and advancements on carrier transport in QD films [20,28,40,41]. Many of the lessons learned by studying other QD devices such as solar cells [42,43,44,45,46,47,48,49,50,51] and, in the case of visible emitting QDs, light-emitting diodes (LEDs) [52,53,54,55] can be applied to QD photodetectors because the key point of focus in many of these studies was enhancing carrier mobility and collection.

Low carrier mobility in QD films, which prevents photogenerated carriers from being collected before recombining with the opposite carrier or with a “trap” state, continues to be a pressing issue for the improvement of QD devices [28]. QD films initially suffered from very poor charge transport because of long insulating ligands [54,56,57]. Colloidally synthesized QDs are stabilized in solution by long organic ligands that are attached to the QD surface, which prevent them from precipitating out of solution when in colloidal form but inhibit charge transport when QDs are incorporated into a film. In order to decrease the inter-QD spacing within films, long chain ligands can be either be exchanged with other shorter ligands [19,20,57,58] or stripped off all together [59,60]; often, this is accomplished by application of layer-by-layer deposition techniques. Layer-by-layer ligand exchange is a process in which a QD film is deposited onto a substrate, typically by drop-casting or spin-coating, and then subsequently exposed to a solution of a shorter ligand (e.g., 1,2-ethanedithiol (EDT) in acetonitrile), affecting replacement of native ligands and allowing QDs to come into closer contact. Ligand exchange is only effective for a deposited QD film sufficiently thin to allow for complete solution penetration, and typically results in voids or “cracks” due to the loss of volume previously taken up by longer ligands. Thus, repetition is required, making layer-by-layer deposition laborious and time consuming, particularly for thicker films. In 2017, Lin et al. [61] introduced a universal method for in-solution ligand exchange of PbE QDs with short ionic ligands, allowing for the deposition of thicker films and removal of post deposition ligand exchange step. Regardless of approach, decreased QD–QD spacing improves the mobility of carriers through increased coupling, making it easier for charge transport to occur either by tunneling or thermally assisted carrier hopping [20,28]. It has also been observed that majority carrier type [21] (i.e., *n*-type [62] or *p*-type [63]) as well as the position of valence and conduction band [18] can also be controlled by modifying the surface chemistry of the QDs through ligand exchange, as shown in Figure 1B. 

Altering the positions of energy bands via ligand exchange can open pathways of band-alignment engineering in which a QD film’s energy levels can be altered to better interact with other layers in the device [45], a technique that was used, for instance, to create a “carrier funneling” effect in QD solar cells [43,44]. Modifying the composition of QDs, either on the surface via ligand exchange or “colloidal atomic layer deposition” [64], or internally through impurity doping [65], also leads to significant effects on the electronic transport properties of the QD film. In one example, Figure 1C shows the transition from *p*-type to *n*-type transport with the addition of excess Pb on the surface of lead selenide (PbSe) QD solids, indicating the role of surface stoichiometry on transport characteristics. Gas-phase atomic layer deposition (ALD) of materials such as alumina has been used to great success for improving carrier mobility [66,67], unlocking carrier multiplication in PbE films [23], and improving air stability. In ALD, a substrate is coated with a material one atomic layer at a time by controlled exposure to one or more reactive gas- or vapor-phase precursors. For QD films, the film can be encased by a layer of the material on top of the film, generally enhancing stability, and/or “infilled”, wherein essentially all voids between QDs are filled with the material [66], which produces enhanced QD–QD coupling and correspondingly larger effects on transport. As the extreme case of enhanced mobility, the possibility of band-like transport in highly coupled QD solids is being pursued vigorously, with some promising prospects, including report of carrier mobilities of QD films reaching values [68] of 24 cm^2^/Vs. 

However, these values pale in comparison to those of layered 2D materials such as graphene, which can have carrier mobilities of up to 15,000 cm^2^/Vs [69]. Layered 2D materials have been of interest for optoelectronic devices due to their promising characteristics and physical versatility, since the successful isolation, through micromechanical cleavage, of a single graphene layer in 2004 [70]. Layered materials have strong in-plane bonding but weak layer-to-layer bonding through van der Waals interactions, enabling cleavage or exfoliation into two-dimensional layers of single unit cell thickness [71,72]. Graphene is an especially interesting material for broadband photodetection purposes because it has zero band gap, meaning that it absorbs light over a wide range of photon energies, from the ultraviolet (UV) to far-infrared [73,74,75,76,77]. Additionally, graphene displays ultrafast carrier dynamics [78,79,80], tunable optical properties via electrostatic doping [81,82,83], and high carrier mobility [84,85,86,87]. The high carrier mobility enables ultrafast conversion of photons to electrical currents or voltages [88,89]. However, the lack of a bandgap in graphene makes it difficult to fabricate devices with low dark current and high on/off ratios because of the presence of free carriers. Techniques such as fabricating graphene nanoribbons via nanostructuring [90,91,92], nanopatterning to create a graphene nanomesh [93,94], and chemical functionalization [95,96,97,98] have been used to engineer a bandgap in graphene [99]; however, these processes often lead to diminished mobility and add unwanted steps and cost to the fabrication process.

**Figure 2:**
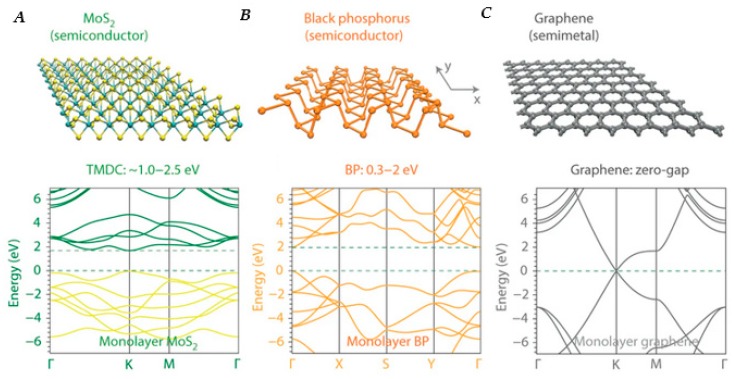
Single-layer atomic structure (top) and band structure (bottom) of selected 2D layered materials of interest. (**A**) Moleybdenum disulfide (MoS_2_). (**B**) Phosphorene (black phosphorous). (**C**) Graphene. Figure (**A**–**C**) Reproduced with permission from [72]. Copyright Springer Nature, 2014.

Although graphene has been at the forefront of layered 2D materials research, transition metal dichalcogenides (TMDs) and other layered 2D materials such as phosphorene [a single 2D layer of black phosphorous (BP)] have also shown promise for potential applications in which a non-zero bandgap is advantageous [72,100]. Phosphorene has a unique 2D structure (see Figure 2B), which causes it to display anisotropic carrier mobility [101], meaning that mobility within the plane is dependent on the direction in which the carrier is traveling. Phosphorene is also highly unique in that it displays *p*-type characteristics [101], and a high work function [102] making it a valuable material for hole transport and hole injection [103]. Figure 2 shows the energy band structures of graphene, phosphorene, and molebdynum disulfide (MoS_2_), a TMD that is one of several that display a transition from indirect to direct bandgap as the sample is reduced from bulk to single monolayers [104,105], which is key for efficient photon absorption [71]. Photodetectors utilizing layered 2D materials have been widely reported [89,103,106,107,108,109,110,111,112], as have studies on charge transport and electronic properties of these materials [113,114,115,116,117,118,119,120]; however, the relatively low absorption, attributable to the atomically thin profile [121], remains a serious challenge.

An attractive option is to combine QDs with layered 2D materials in hybrid devices that allow one to harness many of the desirable features of QDs, including strong, size tunable absorption, long exciton lifetimes and advanced phenomena such as carrier multiplication, while exploiting the excellent charge transport properties of 2D materials. Accordingly, in a typical hybrid QD-2D photodetector, the QD layer acts as the light-absorbing, charge-generating layer, while the 2D layer acts as the transport layer [122], with charge transfer between the layers being a key process for optimization. Such charge transfer affected by various parameters such as ligand length (i.e., QD-2D spacing) and relative band alignment [123], in a similar manner to the role these factors play in charge transfer within QD-only films as discussed above. In this paper, we will briefly review types of photodetectors and their figures of merit and then discuss recent advancements in hybrid QD-layered 2D photodetectors, with emphasis on graphene-QD hybrid and TMD-QD hybrid devices. 

## 2. Types of Photodetectors

### 2.1. Photoconductors

**Figure 3:**
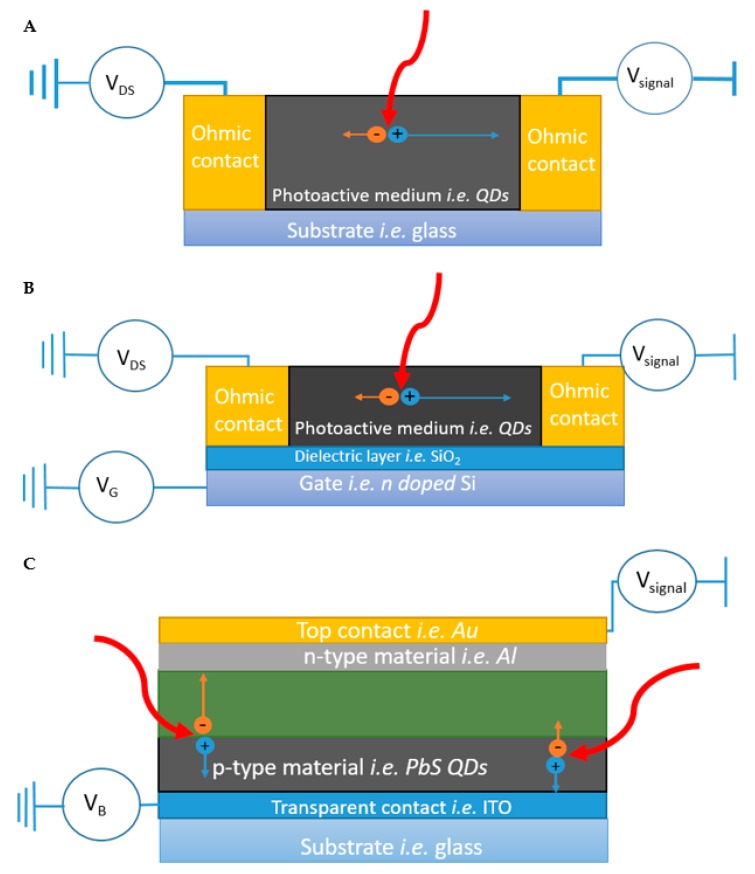
(**A**) Schematic of a photoconductor device. Incident photons [thick red curved arrows] cause electron-hole pairs to form within the photoactive region [black]. A constant applied voltage [*V_DS_*] causes electrons [orange circles] and holes [blue circles] to traverse the photoactive region towards their respective electrodes at different speeds, indicated by the different lengths of orange and blue arrows (in this example holes are traveling faster). As hole are collected at the drain electrode, new holes are injected at the source electrode to maintain charge neutrality; as holes circulate through the device gain is achieved. (**B**) Schematic of a phototransistor device. Operation is similar to a photoconductor, but the device is fabricated on a substrate that allows for the possibility of applying a gate voltage [*V_G_*] to tune transport in the photoactive area. (**C**) Schematic of a photodiode device. A *p*-*n* junction enhances charge transport by creating an internal electric field. The green shaded region indicates the depletion region, which can be tuned by altering the bias voltage [*V_B_*]. Carriers generated in the depletion region are quickly separate and are collected, while carriers generated outside of the depletion region must avoid recombination while they diffuse to either depletion region or contact. All devices are displayed as having a current readout; this signal is typically converted into voltage by a load resistor for easy readout using an oscilloscope.

A photoconductor is a two-contact optoelectronic device in which two ohmic source and drain electrodes are separated by a photoactive layer (i.e., a QD film; Figure 3A). Operated under an applied bias, photoconductors detect temporary changes in the carrier mobility, density, or both under incident illumination due to photogeneration of carriers in the photoactive layer [124]. Under a moderate field, the majority carriers (which can be either holes or electrons, depending on the material) have a higher mobility than the minority carriers. This results in the majority carriers having a shorter transit time to traverse the photoactive area to an electrode, while minority carriers remain left behind. If holes, for example, are the majority carrier, as they are swept out of the detector, charge neutrality is maintained by additional holes supplied from the other electrode. Therefore, effectively, holes can circulate the detector many times during the carrier lifetime, resulting in gain (a measure of how much the response departs from linear dependence) [4,125]. Higher gain leads to detectors with high responsivities, but also higher noise levels which hinders high sensitivity applications [8]. Taking advantage of differential carrier mobility also means photoconductors typically have slower response times than a photodiode, due to the temporal response also being determined by the lifetime of the trapped carriers. Photoconductors are more common for IR applications such as thermal imaging [126] and motion detection [127].

### 2.2. Phototransistors

Phototransistors are essentially photoconductors bridged by a “gate” electrode, typically a metal or degenerately doped semiconductor separated by a dielectric spacer layer. The gate electrode provides the ability to modulate transport using an applied gate voltage; a schematic of a simple phototransistor can be seen in Figure 3B. Phototransistors function in a similar manner as field-effect-transistors (FETs) and are even referred to as optical FETs (OFETs) on occasion [27]. Applying a gate bias introduces charges into the conductive channel, which can be used to tune transport by, e.g., filling trap states [27], as in the case for devices based on *p*-type lead chalcogenide QD films, for which introduction of holes by a negative applied bias increases the conductivity. Phototransistors have become a preferred architecture of the hybrid QD-2D detectors because the level of control offered by the ability to modulate carrier dynamics and concentrations within the device allows for more versatility than a typical photoconductor.

### 2.3. Photodiodes

Photodiodes employ an internal electric field established within the absorbing layer of the device to enhance the efficiency with which photogenerated carriers are separated and subsequently collected. The internal electric field is created by the pairing of materials with majority carriers of opposite charge, which interdiffuse and undergo recombination near the junction to create a depletion region featuring a charge gradient and built-in field. The field favors very fast, unidirectional transport by drift for photogenerated electrons and holes (in opposite directions, respectively), largely preventing recombination. In contrast carriers generated outside the depletion region travel by much slower diffusion to reach either an electrode or the depletion region [125], allowing more time for recombination; accordingly, the size of the depletion region is an important factor in performance. An example of a *p-n* junction photodiode device structure is presented in Figure 3C. Photodiodes are typically operated under moderate reverse bias to create an even wider depletion region to minimize transit time as much as possible, but not too wide or transit-time effects will limit the response frequency [125]. Photodiodes are often categorized by the types of materials forming the junction (e.g., Schottky junction, *p-n* junction, *p-i-n* junction), but the principles of separating and collecting photogenerated charges using a built-in electric field are similar [4], and the relation between device thickness and carrier diffusion length (as determined by carrier lifetime and mobility) is critical to performance. For reference, among QD-based devices, PbE QD solids have exhibited record-high diffusion lengths of up to 230 nm [128]. Photodiodes have a gain of 1 unless operated in avalanche mode, under large reverse biases, where impact ionization and carrier multiplication can result in gains higher than 1. Photodiodes can also be operated under a zero bias condition; however, under reverse bias conditions, the device can have greater bandwidth and wider linear dynamic range. Applications requiring fast response times are heavily reliant on photodiodes [125], as current commercially available photodiodes can have response times as fast as 35 ps. 

## 3. Figures of Merit

Figures of merit [124,125,129] that account for device variables, such as device active area, response time, and spectral region are key for evaluating and comparing detector performance. The first measure of how effective a photodetector can be is how efficiently incident photons are converted into electron-hole pairs and subsequently collected as photocurrent. The external quantum efficiency (EQE) is a measure of how well the device absorbs and converts incident photons into photocurrent, while the internal quantum efficiency (IQE) is a measure of how efficiently absorbed photons are converted into photocurrent. The incident photon flux (*ϕ_in_*) can be calculated by dividing the incident power by the energy of the incident photon. Similarly, the absorbed photon flux (*ϕ_abs_*) can be calculated by multiplying the *ϕ_in_* by the fraction of light that is absorbed [130].

(1) $$EQE\left(\lambda \right)=\frac{\left(\frac{I_{ph}\left(\lambda \right)}{q}\right)}{\phi_{in}\left(\lambda \right)}$$

(2) $$IQE\left(\lambda \right)=\frac{\left(\frac{I_{ph}\left(\lambda \right)}{q}\right)}{\phi_{abs}\left(\lambda \right)}$$

In the preceding equations, *q* is the elementary charge and *I**_ph_*** is the photocurrent, which can be calculated by *I_ph_* = *I_illum_* − *I_dark_,* where *I_illum_* is the photocurrent under illumination and *I_dark_* is the dark photocurrent. The fraction of light absorbed by a QD layer can be calculated with the Beer-Lambert law; for many films this equation can be written in terms of optical thickness (τ), which can be expressed as τ = L $\overset{N}{\underset{i=1}{\sum }}\sigma_{i}n_{i}$, where *σ_i_* and *n_i_* are the attenuation coefficients and number densities of each attenuating species in the absorbing medium. The absorbance can then be calculated with the relationship *A = τ/*ln10. Ellipsometry can also be used to determine refraction indexes and extinction coefficients of materials, which can then be used to create a model for photon absorption [33,131]. 

The responsivity [*R*(*λ*)] of a detector is a measure of the electrical signal output relative to the optical signal input, similar to the EQE. The units of *R(λ)* are amperes per watt [A/W] and it can be calculated by the following equation [124]:

(3) $$R\left(\lambda \right)=\frac{EQE\left(\lambda \right)q\lambda }{hc}\frac{1}{\sqrt{1+\omega^{2}\tau^{2}}}G\left(\lambda \right)$$

where λ is the wavelength of the incident photons, *h* is Planck’s constant, *c* is the speed of light, *ω* is the modulation frequency, τ is the time constant, and *G* (*λ*) is the photoconductive gain. Photodiodes have a gain of 1 unless they are operated in avalanche mode, while for photoconductors and phototransistors the gain is equal to the majority carrier lifetime divided by the transit time [*G*(*λ*) *= τ*_lifetime_*/τ*_transit_]. Transit time can be calculated from the carrier mobility and bias voltage by *τ*_transit_ *= L*^2^/(*μV*_bias_) [130]. 

Noise equivalent power [*NEP*(*λ*)] is a measure of the detector sensitivity, defined as the optical power at which the signal-to-noise ratio (SNR) is 1, giving the minimum power the detector can effectively detect per square root of bandwidth [124], and is obtained using:

(4) $$NEP\left(\lambda \right)=\frac{\sqrt{\bar{I_{n}^{2}}}}{R\left(\lambda \right)}$$

where *I_n_* is the total noise current, and *NEP* (*λ*) is in units of W/Hz^1/2^. Total noise current is the sum of all the noise sources, which includes low-frequency flicker noise (1*/f*), thermal noise (*I*_th_) and shot noise (*I*_sh_). The root-mean-square value of thermal noise and shot noise current is given by *I*_th_ *=* $\sqrt{4kTB/r}$ and *I*_sh_ *=* $\sqrt{2qBI_{dark}}$*,* respectively, where *k* is Boltzmann’s constant, *T* is absolute temperature, *r* is the resistive element source contributing to noise, *B* is the noise bandwidth, and *I*_dark_ is the dark current [3].

The most useful figure of merit is the detectivity [D*(*λ*)], which accounts for different configurations and detector areas [3], allowing for comparison across all devices. The D*(*λ*) of a photodetector is expressed in units of Jones (cm Hz^1/2^/W), and is obtained using the following equation:

(5) $$D^{*}\left(\lambda \right)=\frac{\sqrt{A}}{NEP\left(\lambda \right)}$$

where *A* is the detector active area (cm^2^). D*(*λ*) is proportional to *R*(*λ*), and it is also an indirect function of applied bias, temperature, modulation frequency, and wavelength [3,124]. When reporting D*(*λ*) the value should be accompanied by the measurement conditions to ensure that presented values are both correct and can be properly used for comparison of multiple devices.

The linear dynamic range [*DR* (*λ*)] shows the range over which the photocurrent increases linearly with increasing incident optical power and provides for the range of optical power over which detector should be utilized. Ideally, *R* (*λ*) should remain constant with the increase of optical intensity. *DR* (*λ*) has units of decibels (dB) and is expressed as:

(6) $$DR\left(\lambda \right)=20\log\frac{P{\left(\lambda \right)}_{\max}}{NEP\left(\lambda \right)}$$

where *P*(*λ*)_max_ is the incident optical power when the photocurrent saturates [124].

## 4. Graphene-QD Hybrid Detectors

As described above, graphene has been an attractive material for photodetection applications since its discovery, due to high carrier mobilities and potential broad spectrum applicability [130], and QDs are of interest because of strong, tunable light absorbance. Sensitizing graphene-based photodetectors with PbE QDs can improve D* of a device by improving absorption in the short-wave IR and visible regions. Photon absorption in a layer of QDs creates electric charges that can then be transferred to the graphene for fast transport. In the case of phototransistors and photoconductors, the high carrier mobility combined with the long carrier lifetimes in the QD layer would potentially allow the carriers to circulate the device many times before recombining, leading to high gains.

**Figure 4:**
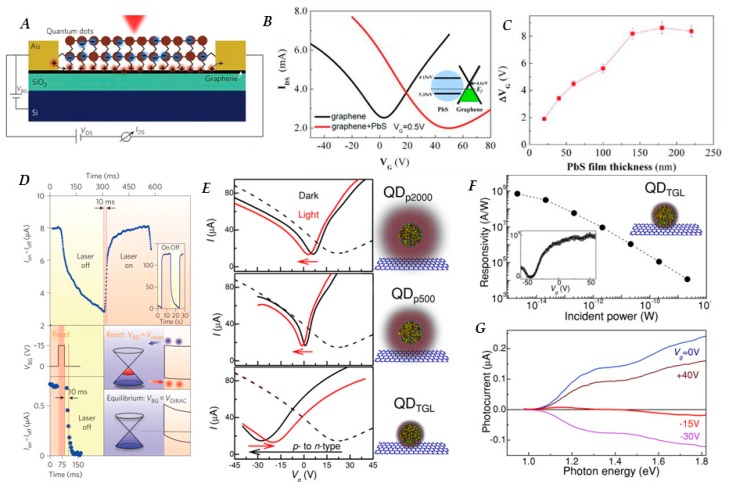
(**A**) Schematic representation of a typical graphene-QD hybrid phototransistor, in which graphene acts as gate modulated transport layer for holes; (**B**) Transfer characteristics (source-drain current (*I_DS_*) ~ *V_G_*, V_DS_ = 0.5 V) of bare un-doped graphene transistors before (black) and after the deposition of PbS QDs on the graphene film (red). Indication of *p-*type doping in the graphene film is shown by the transfer curve becoming asymmetric and the Dirac point shifting to a positive gate voltage (~50 V) after deposition. Inset: energy diagram of the heterojunction of PbS QD and graphene (valence and conduction band values of the PbS QD are 5.35 eV and 4.15 eV respectively, while the value for graphene reads 4.6 eV)***;*** (**C**) Horizontal shift of the transfer curves (*I_DS_* ~ *V_G_*, *V_DS_* = 0.5 V) of the hybrid graphene-PbS QDs devices with different thicknesses of PbS QDs layers under irradiation with 6.4 mW cm^−2^ of 895 nm light. Saturation after 150 nm indicates that any carriers generated further than 150 nm from graphene layer are not collected; (**D**) [Top] Photocurrent response as a function of time of a hybrid graphene-PbS QDs phototransistor. The temporal response indicates a rise time of ~10 ms, and two different fall times on the order of 100 ms (50%) and 1s. Inset, measured at a higher power of 267 pW; [Bottom] temporal response of a bilayer graphene phototransistor after the laser is turned off and application of a reset pulse for 10 ms. The fall time is reduced from several seconds to ~10 ms. Insets: energy diagrams showing effect of reset pulse lowering potential barrier allowing electrons trapped at graphene-QD barrier to escape; (**E**) Transfer characteristics (*I_DS_*-*V_G_*_,_ *V_DS_* = 0.1 V) of *p*-doped graphene phototransistors before (dashed lines) and after (black) deposition of PbS QD with varying ligand length (schematic not to scale) red line is transfer curve under illumination with unfocused laser light (λ = 514 nm) with *P* = 10 W m^−2^. Size of shift after deposition is an indication of coupling between graphene and QD layer; shift upon illumination is an indication of charge transfer of photoexcited carriers. (**F**) Responsivity as a function of incident power for hybrid detector using short thioglycerol (TGL) ligands. Inset: responsivity as a function of *V_G_*; (**G**) Photocurrent dependence on photon energy of the incident light (P ≈ 10^−11^ W) at different gate voltages for same device as Figure (**F**). Figure (**A**,**D**) reproduced with permission from [122]. Copyright Springer Nature, 2012. Figure (**B**,**C**) reproduced with permission from [132]. Copyright John Wiley and Sons, 2012. Figure (**E**–**G**) reproduced with permission from [133]. Copyright John Wiley and Sons, 2015.

In 2012, Konstantatos et al. [122] reported a hybrid graphene/PbS QD phototransistor with ultrahigh gain, sparking much interest in the system and inspiring many variations to be fabricated in the following years. In the seminal device, single and bilayer graphene flakes were prepared by mechanical exfoliation and deposited on Si/SiO_2_ substrates, followed by gold contact deposition and, finally, PbS QD deposition via spin-coating. Ligand exchange from oleic acid to EDT was performed during the QD deposition phase through a layer-by-layer approach, with a resulting film thickness of ≈80 nm. A schematic of the device can be seen in Figure 4A. The photo-generated holes in the PbS QD layer are transferred to the graphene layer while the photo-generated electrons remain trapped in the PbS QD layer. Due to the high hole mobility in graphene, these carriers are allowed to circulate in the device, while charge conservation in the graphene channel is enabled by hole replenishment from the source electrode for every hole collected at the drain, resulting in a gain of 10^8^ electrons per photon. The vast improvement of the gain led to an *R* on the order of 10^7^ A/W and a D* of 7 × 10^13^ Jones. A short gate voltage pulse can be used to purge the charge carriers from the QD layer as shown in Figure 4D, effectively resetting the device and increasing the operating speed, which is advantageous for imaging applications.

Later in the same year, Sun et al. [132] used chemical vapor deposition (CVD) rather than mechanical exfoliation to prepare the graphene transport layer. CVD allows for precise control over thickness, as well as the possibility of fabricating larger area devices than what is feasible with mechanically exfoliated graphene. Upon deposition of PbS QDs capped with pyridine ligands onto un-doped graphene, the measured Dirac point shifts from 0 to 50 V, indicating *p*-type doping of the graphene layer, as can be seen in Figure 4B. Along with this shift in the Dirac point, there is a sizable decrease in electron mobility from 1000 cm^2^/Vs to 440 cm^2^/Vs while the hole mobility remained unchanged at 1000 cm^2^/Vs. Responsivity of the detector was found to be affected by the thickness of the QD layer, with a saturation point occurring at ≈150 nm, as can be seen in Figure 4C. The devices using CVD-grown graphene showed *R* values of 1 × 10^7^ A/W, similar to that of detectors using mechanically exfoliated graphene.

Ligand length has a significant effect on the efficiency of charge transfer from QDs to graphene [133], as can be seen in Figure 4E by the more significant shift in the Dirac point of the graphene/QD system when a shorter capping ligand is utilized, indicating increased coupling between graphene and QDs. Figure 4F shows the responsivity of the QD/graphene system can reach values on the order of 1 × 10^9^ A/W with shorter capping ligands such as thioglycerol (TGL) (length 0.5 nm). Ideally, photocurrent as a function of incident photon energy of devices should closely follow the absorption features of the QDs used, as seen in Figure 4G, indicating increased sensitivity in the range where QDs are strongly absorbing. Laser shock imprinting has also been used as a method to improve contact between graphene and QDs in graphene/PbS QD/graphene sandwich structure [134], resulting in improved response time and current on/off ratio.

Multi-heterojunction phototransistors synthesized by spin-coating alternating graphene and PbSe QD layers show the importance of using graphene as the bottom layer of the device [135], with graphene bottom layer device displaying electron and hole mobilities of *μ_E_* = 147 cm^2^/Vs and *μ_H_* = 137 cm^2^/Vs, while QD bottom layer devices showed *μ_E_* = 14 cm^2^/Vs and *μ_H_* = 59 cm^2^/Vs. Intercalation of graphene layers within a PbS QD film also improves the charge carrier extraction of the device by counteracting the limitation of diffusion length in QD films. Placing graphene layers, separated by a distance less than the carrier diffusion length of the QD film, periodically through a QD/graphene film results in higher photocurrents than devices with only a bottom graphene layer [136].

Electrohydrodynamic nanoprinting of colloidal PbS QDs onto graphene FETs with varying quantum dot layer thicknesses is a potential method for realizing small footprint detectors with high spatial resolution [137]. The responsivity of the photodetectors increases with increasing layer thicknesses up to 130 nm. However, the noise current is found to be independent of the layer thickness. Additionally, responsivity and noise current are both linearly dependent on the applied drain voltage and drain current. As a result, the specific detectivity is independent of the drain voltage, and the detector can be operated at lower drain voltage thus reducing power consumption. D* values of at least 10^9^ Jones are reported without degradation of the charge carrier mobilities in graphene from the electrohydrodynamic printing of QDs [137].

**Figure 5:**
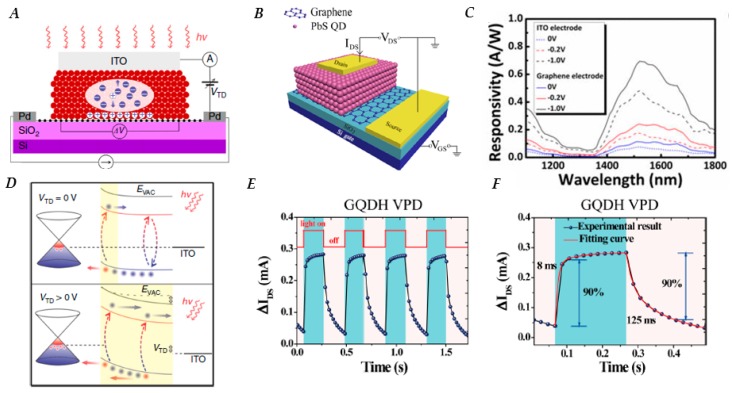
(**A**) Schematic representation of a combined QD photodiode and graphene phototransistor device; *V_TD_* creates a bias in the photodiode. (**B**) Schematic of a typical hybrid graphene-QD vertical phototransistor using graphene as an electrode. (**C**) Comparison of responsivity as a function of wavelength at various applied biases for devices using graphene and indium tin oxide (ITO) as an electrode, respectively. (**D**) Energy band diagram of the graphene-QD interface; yellow shading indicates the depletion region in the QD layer. Top schematic is when detector is operated in only phototransistor mode, while the bottom shows the expansion of the depletion region when QD layer is used as a photodiode with an applied bias. (**E**) Photocurrent response of hybrid graphene-PbS QD phototransistor as a function of time for light on/off cycles at an irradiance of 335 mW/cm^2^, (*V_DS_* = 1 V and *V_GS_* = 1.5 V). (**F**) Zoomed in view of Figure (**E**) to see rise and decay times of device, rise time 8 ms, decay time 125 ms. Figure (**A**,**D**) reproduced with permission from [138]. Copyright Springer Nature, 2016. Figure (**B**) reproduced with permission from [141]. Copyright AIP Publishing, 2016. Figure (**C**) reproduced with permission from [140]. Copyright AIP Publishing, 2011. Figure (**E**,**F**) reproduced with permission from [142]. Copyright American Chemical Society, 2017.

Response time of phototransistors with thicker QD films (>100 nm) is still regulated by the diffusion of carriers through the QD sensitizing layer. However, this can be overcome by a device architecture that combines a graphene–colloidal QD photodiode and a high-gain phototransistor; a schematic of the devices can be seen in Figure 5A. Transforming the electrically passive sensitizing layer to an active one through an applied electric field in the photodiode significantly enhances charge collection, due to carrier drift instead of solely relying on diffusion [138]. The QD photodiode consists of a top-contact (e.g., indium tin oxide (ITO)) acting as the cathode of the QD photodiode, whereas graphene acts as the hole acceptor contact and the charge transport channel for the phototransistor. Progressively increasing the bias voltage causes the depletion region to grow, enhancing efficiency of the charge collection (Figure 5D). The hybrid device architecture results in a sub-millisecond temporal response, a gain-bandwidth product on the order of 10^8^, a linear dynamic range in excess of 110 dB, and very high sensitivity with experimentally measured D* of 1 × 10^13^ Jones.

In addition to the properties described above, graphene’s weak electrostatic screening effect, finite density of states and mechanical flexibility [139] makes it a versatile material for conducting electrodes. Due to its relative transparency at wavelengths greater than 1000 nm, compared to ITO [140], there has been a push for using graphene as the transparent conducting electrode in IR detection and imaging applications. Responsivity of graphene-based and ITO-based PbS QD photodiodes from wavelengths of 1100–1800 nm operated at different reverse bias conditions are shown in Figure 5C. The responsivity of graphene-based vs. ITO-based PbS photodiodes are 0.112 and 0.076 A/W at 1530 nm, respectively, and increase to 0.69 and 0.50 A/W at bias of −1 V. Photocurrent response of the devices under IR illumination (1530 nm) was found to increase linearly with light power. Ambipolar vertical phototransistors (Figure 5B) [141] utilizing graphene as an electrode have been fabricated using both PbS [142] and PbSe [139] QDs. In a vertical phototransistor, the channel length is determined by the film thickness, which is much shorter than a typical lateral phototransistor channel, leading to faster response times. Vertical phototransistors utilizing graphene as source electrode with PbS QDs displayed temporal response times of 14 ms, which improves to 8 ms when graphene is mixed within the QD layer, as seen in Figure 5E,F, while PbSe QD detectors showed a response time of 7 ms.

## 5. Merging QDs with TMDs and Other Layered 2D Materials

**Figure 6:**
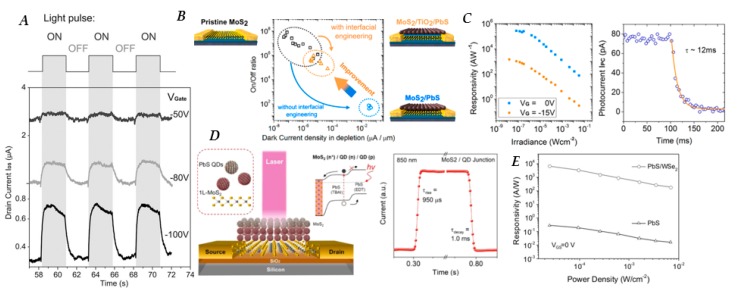
(**A**) Photocurrent response of hybrid molebdynum disulfide (MoS_2_)-PbS QD phototransistor as a function of time at various, relatively high gate voltages (*V_DS_* = 1 V, irradiance 3 µW/cm^2^). (**B**) Schematics of bare MoS_2_ phototransistor as well PbS QD/MoS_2_ phototransistor with and without titanium(IV) oxide (TiO_2_) buffer layer; label colors correspond with points on the graph. Graph shows on/off ratio as a function of dark current density, demonstrating that a TiO_2_ buffer layer helps to reduce dark current. (**C**) [Right] Responsivity as a function of irradiance for hybrid MoS_2_-PbS QD device with a TiO_2_ buffer layer at different gate voltages (*V_DS_* = 1 V). [Left] Photocurrent decay time of a light response at 67 nW/cm^2^; the approximation with a single-exponential function results in a time constant of 12 ms. (**D**) Schematic of hybrid MoS_2_-PbS QD devices using 1,2-ethanedithiol (EDT) and tetrabutylammonium iodide (TBAI) ligands to create a built in potential (energy-band diagram inset), and [right] response time of device under laser 850 nm laser illumination (*P* = 200 nW, *V_DS_* = 1 V_,_ *V_G_* = 0 V). (**E**) Responsivity as a function of power density of PbS QD and hybrid tungsten diselenide (WSe_2_)-PbS QD phototransistors under 970 nm illumination (*V_DS_* = 1 V, *V_G_* = 0V). Figure (**A**) reproduced with permission from [143]. Copyright John Wiley and Sons, 2015. Figure (**B**,**C**) reproduced with permission from [144]. Copyright American Chemical Society, 2016. Figure (**D**) reproduced with permission from [145]. Copyright American Chemical Society, 2018. Figure (**E**) reproduced with permission from [147]. Copyright John Wiley and Sons, 2017.

While graphene-QD hybrid detectors display high gain, they suffer from high dark currents due to the semi-metallic nature of graphene. Transition metal dichalcogenides (TMDs) have relatively large bandgaps (1–2.5 eV) [72], which make them interesting candidates for applications that require high sensitivity. Using 2D TMDs rather than graphene presents a trade-off in carrier mobility and ultimately gains in exchange for lower dark conductivities. In 2015 Kufer et al. [143] published the first hybrid MoS_2_-PbS QD photodetector, in which micromechanically exfoliated MoS_2_ nanosheets were used as electrically controllable transport layers, resulting in responsivities on the order of 6 × 10^5^ A/W. D* of bilayer and few-layer devices were found to be 2 × 10^11^ and 5 × 10^11^ Jones, respectively. At high negative back-gate bias the MoS_2_ channel is depleted from free carriers in the dark state, giving the detector the potential to reach high sensitivity in the shot noise limit with D*_shot-noise limit_ reaching up to 7 × 10^14^ Jones at *V_G_* of −100 V (Figure 6A). Application of a semiconducting titanium(IV) oxide (TiO_2_) buffer layer at the interface of MoS_2_ and PbS QDs, as presented in Figure 6B, preserves the gate modulation by suppressing the high density of localized sub-band-gap states that pin the Fermi level [144]. The maintained gate control over carrier density in the conduction channel allows for low noise operation similar to pristine MoS_2_ devices, resulting in a D* of 5 × 10^12^ Jones, an improvement of more than 1 order of magnitude compared to MoS_2_/PbS devices without a buffer layer [144]. Figure 6C shows the responsivity as a function of irradiance as well as the decay time of MoS_2_/PbS devices when a TiO_2_ buffer layer is utilized.

Applying methods that were previously used in QD-only devices to provide a built-in *p*-*n* junction via energy level modification through ligand engineering can also have positive impacts on hybrid devices. The combination of tetrabutylammonium iodide (TBAI) and EDT is a well-known ligand combination used to create such a built-in potential in QD photovoltaics, resulting in more efficient charge carrier separation in the QD layer. Combining a layer of EDT-treated PbS QD with a layer of TBAI-treated PbS QD along with and MoS_2_ transport layer in a vertical phototransistor, as shown in Figure 6D, resulted in fast response times (960 µs), and D* on the order of 10^11^ Jones under applied gate voltage of −40 V [145].

The range of TMD materials used in hybrid PbE QD photodetectors has also been expanded to include tungsten disulfide (WS_2_) [146] and tungsten diselenide (WSe_2_) [147] which show higher carrier mobilities than MoS_2_. Phototransistors fabricated using CVD to fabricate a *p*-type WSe_2_ monolayer on Si/SiO_2_ substrate, coated with PbS QDs with TBAI ligands, displayed rise times of 7 ms and a decay time of 480 ms. The responsivity of hybrid WSe_2_-PbS device could be tuned by the applied gate voltage, but operating in depletion mode was not necessary, which overcomes a drawback for the MoS_2_-QD hybrid device. The highest D* of the device was found to be 7 × 10^13^ Jones, with a responsivity of up to 2 × 10^5^ A/W, as seen in Figure 6E. WS_2_/EDT-capped PbS QD devices were fabricated in a similar manner resulting in rise and decay times of 153 µs and 226 µs, respectively; however, the responsivity (14 A/W), and D* (3.9 × 10^8^ Jones) of the devices were lower than the WSe_2_ counterpart. Detectors utilizing both WS_2_ and MoS_2_ combined with larger (8.0 ± 1.7 nm diameter) PbS QDs, with an absorption peak near 1.8 μm show compelling results with responsivities of 1400 A/W, at 1.8 μm excitation operated at 1 V bias, and D* as high as 10^12^ Jones at room temperature for the WS_2_ based devices [148]. The devices employing larger dots showed better results using Zn_2_I and mercaptopropionic acid (MPA) ligands, rather than the traditional EDT ligand, as this ligand combination has been shown to produce higher mobilities in larger PbS QD films [149].

**Figure 7:**
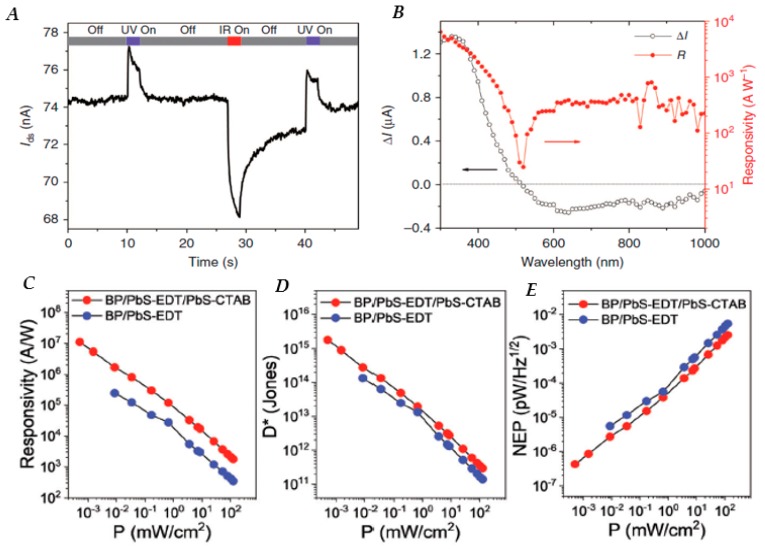
(**A**) Photocurrent as a function of time under illumination with 365 and 970 nm light-emitting diode (LED) light sources, showing the spectrally distinctive characteristics of the tin disulfide (SnS_2_)/PbS QD photodetector. (**B**) Wavelength-dependent photocurrent and responsivity of a SnS_2_/PbS QD device; the light source is a xenon lamp modulated with an optical grating to generate monochromatic light with a step-size of 10 nm (*V_G_* = 0 V, *V_DS_* = 1 V). (**C**) Responsivity, (**D**) Detectivtiy and (**E**) noise equivalent power (NEP) of hybrid black phosphorous (BP)/PbS QD devices with EDT only and EDT/cetyltrimethylammonium bromide (CTAB) ligand combinations as a function of power intensity at 633 nm wavelength at *V_DS_* = 1 V and *V_G_* = 0 V. Figure (**A**,**B**) reproduced with permission from [150]. Copyright Springer Nature, 2016. Figure (**C**–**E**) reproduced with permission from [153]. Copyright Royal Chemical Society, 2019.

Other layered 2D materials have also been used in hybrid 2D-PbE QD photodetectors. Tin disulfide (SnS_2_)/PbS QD hybrid photodetectors show distinct photoresponse towards photons of different wavelengths [150]. Mechanically exfoliated SnS_2_ nanosheets (5 layers) sensitized with EDT-capped PbS QDs and gold electrodes, yielded a broadband, spectrally distinctive photodetector which displays positive photocurrent at wavelengths below 520 nm (the cutoff absorption wavelength of SnS_2_) and negative photocurrent at wavelengths above 520 nm, as shown in Figure 7A,B. This spectral selectivity is accounted for by illumination-modulated barrier height between the gold electrode and the SnS_2_ nanosheet. Upon NIR illumination, only PbS QDs are absorbing incident photons, and photogenerated electrons flow into SnS_2_ nanosheets, reversing the *p*-type doping effect in the dark and shifting the Fermi level of SnS_2_ nanosheets upwards. Consequently, the contact between SnS_2_ nanosheets and Au electrodes is Schottky in nature, accounting for the observed negative photoconductivity. Under UV illumination, SnS_2_ nanosheets absorb incident photons, resulting in carrier density increasing in the SnS_2_ channel, which overrides the contribution from photogenerated carriers in the PbS QDs, leading to the observed positive photoconductivity [150].

The 2D black phosphorous (BP) nanosheets, also called “phosphorene”, have also recently emerged as a potential candidate for photodetection devices due to high carrier mobilities and anisotropy. Phosphorene has typically suffered from formation of phosphoric acid on the surface under ambient conditions, which causes device performance to degrade. However recent work has shown that treatment with EDT recovers the desirable properties of the device even after degradation [151]. Since EDT is also a common ligand treatment for QD devices as well, Lee et al. decided to combine phosphorene and PbS QDs into a hybrid photodetector, resulting in responsivities of 5.36 × 10^8^ A/W and a D* of 1.89 × 10^16^ Jones [152]. A hybrid BP/PbS QD photodetector with a cascade-type energy band structure can be fabricated by using ligand chemistry to alter the energy bands of two different layers of PbS QDs. EDT and cetyltrimethylammonium bromide (CTAB) ligands are utilized to form an additional energy barrier at the interface of bilayer QDs. A high responsivity of 1.1 × 10^7^ A/W, a D* of 1.75 × 10^15^ Jones and a low noise equivalent power of 4.3 × 10^7^ pW/Hz^1/2^ are achieved at a bias of 1 V without gate voltage modulation, as shown in Figure 7C,D,E, respectively. The responsivity is an order of magnitude higher compared to the phosphorene/PbS photodetector that used only EDT ligands [153]. PbSe QDs have also been integrated with 2D Bi_2_O_2_Se, a 2D material with a relatively narrow bandgap around 0.8 eV, in devices showing impressive responsivities on the order of 10^3^ A/W, when excited with 2000 nm excitation and operated under 100 V bias [154]. Table 1 lists the devices discussed within this review along with their figures of merit.

## 6. Outlook

Hybrid PbE QD-layered 2D photodetectors display great promise for improving detector capabilities, especially in the spectral regions beyond the 1 µm bandgap of Si-based detectors, with much progress made in recent years. However, there are still many avenues for continued progress in improving the figures of merit. There exists a wide and expanding array of studies of PbE QDs aimed at: controlling synthesis to improve size distribution and stability [155,156,157,158]; QD size and composition effects on the optoelectronic properties [39,49,159]; in-solution and layer-by-layer ligand exchange techniques [18,61]; and inter- and intra-layer charge transfer in QD films with other materials [28,45,160]. Many of these findings have yet to be applied to hybrid QD/2D material hybrid devices. Particularly promising directions would involve application of less-commonly used ligand/surface treatments [18,61], expanding the spectral range further into the IR by increasing QD size [13,148], and use of ALD to improve mobility [66,67]. Research and development of new layered 2D materials and techniques for improvement in synthesis of current materials is also an ongoing direction. Strides made in recent years show the great potential of PbE QD-layered 2D hybrid photodetectors on account of facile operation at room temperature, low cost, flexible substrate compatibility, and high figures of merit. Continued progress in the field is possible through continued research, implementation of new materials, surface treatments, and device engineering.

## Figures and Tables

**Table 1:** Hybrid PbE QD-layered 2D photodetectors and their corresponding figures of merit. All detectors presented in this table are phototransistors, with one detector that from [138] utilizing a hybrid phototransistor/photodiode geometry. EDT = ethanedithiol, TBAI = tetrabutylammonium iodide, TGL = thioglycerol, DTG = 2, 3-dimercapto-1-propanol, MPA = mercaptopropionic acid, and CTAB = cetyltrimethylammonium bromide.

QD/Material	Ligand (s)	Excitation Wavelength (nm)	Response (Rise) Time (ms)	Responsivity (A/W)	Detectivity (Jones)	Ref.
PbSe/Graphene	EDT	808	12,000	10^6^	N/A	[135]
PbS/Graphene	TBAI	532	3000 (decay)	5.9 × 10^7^	N/A	[136]
PbS/Graphene	EDT	1200	100	8 × 10^3^	10^9^	[137]
PbS/Graphene	Pyridine	895	130	10^7^	N/A	[132]
PbS/Graphene	EDT	532	10	10^7^	7 × 10^13^	[122]
PbS/Graphene	TGL/DTG	532	N/A	10^9^	N/A	[133]
PbS/Graphene	EDT	635	1	2 × 10^6^	10^13^	[138]
PbS/MoS_2_	EDT	635	350 (decay)	6 × 10^5^	5 × 10^11^	[143]
PbS/MoS_2_	TBAI/EDT	850	0.95	5.4 × 10^4^	10^11^	[145]
PbS/WS_2_	EDT	808	0.153	14	3.9 × 10^8^	[146]
PbS/MoS_2_/TiO_2_	EDT	635	12	10^5^	5 × 10^12^	[144]
PbS/WSe_2_	TBAI	970	7	2 × 10^5^	7 × 10^13^	[147]
PbS/WS_s_	Zn_2_I/MPA	1800	200	1442	10^12^	[148]
PbS/MoS_2_	Zn_2_I/MPA	1800	32	202	2.8 × 10^11^	[148]
PbS/SnS_2_	EDT	970	160 (decay)	10^6^	2.2 × 10^12^	[150]
PbS/BP	EDT	405	770 (decay)	5.36 × 108	1.89 × 10^16^	[152]
PbS/BP	EDT/CTAB	633	N/A	1.1 × 10^7^	1.75 × 10^15^	[153]
PbSe/Bi_2_O_2_Se	EDT	2000	4	10^3^	N/A	[154]
